# Cancer-specific T cell receptor isolation for cancer immunotherapy

**DOI:** 10.1186/2051-1426-3-S2-P380

**Published:** 2015-11-04

**Authors:** Debbie Emma Baker, Linda Hibbert, Luise Weigand, Samantha Paston, Ruth Ryan, Zoe Donnellan, Ruth Simmons, Kathy Hale, Louise Conlon, Joseph Dukes, Vanessa Clark, Caroline Boudousquie, Giovanna Bossi, Emma Hickman, Alex Powlesland, Annelise Vuidepot, Namir Hassan, Bent Jakobsen

**Affiliations:** 1 Immunocore Ltd., Abingdon, UK

## Background

Malignant cells may be recognised by T cells binding cell surface Class I HLA (Human Leukocyte Antigen)-peptide complexes presenting disease-associated epitopes. Many cancer patients have been shown to generate CD8 cytotoxic T cell responses to tumour-associated antigens. However, this is often insufficient for the immune system to clear tumours, resulting in the progression of cancer. This is in part due to the low avidity of these T cells, as well as the ability of cancer cells to develop escape mechanisms to avoid destruction by T cells.

## Methods

To overcome these issues, we have engineered novel, bi-functional protein therapeutics termed ImmTACs (Immune mobilising monoclonal TCR Against Cancer) which re-direct the immune system to target and destroy tumour cells with a high degree of potency and specificity. An ImmTAC comprises a high affinity ‘monoclonal’ T cell receptor (mTCR) targeting a cancer-associated HLA-peptide complex, fused to an anti-CD3 scFv domain which activates an anti-tumour T cell response.

## Results

We have developed an integrated in-house process leading to the isolation of TCRs specific for validated cancer epitopes forming the starting material for ImmTAC production. The essential steps in this procedure are: antigen selection, epitope identification, T cell cloning, TCR isolation and binding to soluble peptide:MHC on the Biacore. High affinity ImmTACs are then generated through a process of affinity maturation.

## Conclusions

The steps leading to the cloning of wild type TCRs are presented here, together with exemplary data to illustrate the successful isolation of TCRs resulting from this process.

